# Combining serum inflammation indexes at baseline and post treatment could predict pathological efficacy to anti‑PD‑1 combined with neoadjuvant chemotherapy in esophageal squamous cell carcinoma

**DOI:** 10.1186/s12967-022-03252-7

**Published:** 2022-02-02

**Authors:** Xinke Zhang, A. Gari, Mei Li, Jierong Chen, Chunhua Qu, Lihong Zhang, Jiewei Chen

**Affiliations:** 1 State Key Laboratory of Oncology in South China, Collaborative Innovation Center for Cancer Medicine, Sun Yat-Sen University Cancer Center, 651#Dong Feng Road East, Guangzhou, 510060 People’s Republic of China; 2 Department of Pathology, Sun Yat-Sen University Cancer Center, Guangzhou, 510060 China

**Keywords:** Serum, Inflammation indexes, Pathological efficacy, Anti-PD-1, ESCC

## Abstract

**Background:**

The neutrophil-to-lymphocyte ratio (NLR), lymphocyte-to-monocyte ratio (LMR), platelet-to-lymphocyte ratio (PLR), and systemic immune-inflammation index (SII) have been used to predict therapeutic response in different tumors. However, no assessments of their usefulness have been performed in esophageal squamous cell carcinoma (ESCC) patients receiving anti‑PD‑1 combined with neoadjuvant chemotherapy.

**Methods:**

The respective data of 64 ESCC patients receiving anti‑PD‑1 combined with neoadjuvant chemotherapy were analyzed. Whether NLR, LMR, PLR, and SII at baseline and post-treatment might predict pathological response to anti‑PD‑1 plus neoadjuvant chemotherapy, and cutoff values of these parameters were all determined by ROC curve analysis.

**Results:**

NLR (cutoff = 3.173, AUC = 0.644, 95% CI 0.500–0.788, P = 0.124, sensitivity = 1.000, specificity = 0.373), LMR (cutoff = 1.622, AUC = 0.631, 95% CI 0.477–0.784, P = 0.161, sensitivity = 0.917, specificity = 0.137), PLR (cutoff = 71.108, AUC = 0.712, 95% CI 0.575–0.849, P = 0.023, sensitivity = 1.000, specificity = 0.059), and SII at baseline (cutoff = 559.266, AUC = 0.681, 95% CI 0.533–0.830, P = 0.052, sensitivity = 0.373, specificity = 1.000) seemed to be a useful predictor for distinguishing responders from non-responders. Combining NLR with SII at baseline (AUC = 0.729, 95% CI 0.600–0.858, P = 0.014, sensitivity = 0.917, specificity = 0.510), LMR and SII at baseline (AUC = 0.735, 95% CI 0.609–0.861, P = 0.012, sensitivity = 1.000 specificity = 0.471), PLR and SII at baseline (AUC = 0.716, 95% CI 0.584–0.847, P = 0.021, sensitivity = 1.000 specificity = 0.431), and LMR and PLR at post-treatment in the third period (AUC = 0.761, 95% CI 0.605–0.917, P = 0.010, sensitivity = 0.800, specificity = 0.696) might slightly increase the prediction ability to determine patients who have response or no response. Finally, combining LMR at baseline, SII at post-treatment in the second period with PLR at post-treatment in the third period could be considered a better predictor for discriminating responders and non-responders than single or dual biomarkers (AUC = 0.879, 95% CI 0.788–0.969, P = 0.0001, sensitivity = 0.909, specificity = 0.800).

**Conclusions:**

The models we constructed allowed for the accurate and efficient stratification of ESCC patients receiving anti-PD-1 plus chemotherapy and are easily applicable for clinical practice at no additional cost.

## Introduction

Esophageal carcinoma (EC) is one of the most common malignancies and the sixth leading cause of cancer deaths worldwide, with an overall 5-year survival rate ranging from 0 to 10% [1, 2]. Pathological subtype of EC includes esophageal squamous cell carcinoma (ESCC) and adenocarcinoma (EAC). At present, the incidence and mortality rates of ESCC are more prevalent in East Asian countries, especially in China. Unfortunately, surgical treatment alone is not satisfactory because most of patients diagnosed are at the locally advanced stage of the disease [3]. Standard neoadjuvant therapy mainly includes neoadjuvant chemoradiotherapy (NRCT) [4, 5] and neoadjuvant chemotherapy (NCT) [6, 7] in locally advanced ESCC, and the NCRT group has the most common significant hematologic toxic effects [8]. The NCT02395705 trial showed that the pathological complete response (pCR) rate of the NCT group was only 10.2% [9], ESCC patients receiving NCT showed poor survival, and over 20% of patients relapsed for locally advanced ESCC [10]. Therefore, more effective treatments are required to improve therapeutic efficacy and clinical outcomes in patients with locally advanced ESCC. It is worth mentioning that a recent phase I study of JCOG1804E (FRONTiER Trial, NCT03914443) was conducted to evaluate the safety of nivolumab as a human monoclonal antibody targeting PD-1 in combination with chemotherapy of CDDP+ 5-FU (CF) or Docetaxel (DTX) + CF (DCF) as neoadjuvant therapy and could provide a new promising neoadjuvant therapy regimen for patients with locally advanced EC [11]. In addition, a preclinical study suggested that an immune checkpoint inhibitor (ICI) as a neoadjuvant obtained better efficacy than that of an adjuvant [12]. This is consistent with other studies reporting that nivolumab monotherapy showed a pCR of 43% in patients with resectable non-small-cell lung cancer [13], though the rate was 20% in lung cancer patients with metastatic disease when nivolumab was considered as an adjuvant [14]. Such a neoadjuvant regimen might strengthen the systematic preparation of anti-tumor T cells, thus potentially eliminating micro-metastatic tumor cells that might lead to postsurgical recurrence. In patients with metastatic ESCC, the Phase II ATTRACTION-1 and Phase III ATTRACTION-3 (NCT02569242) trial demonstrated promising efficacy and safety of nivolumab [15, 16]. Therefore, our study aimed to evaluate pathological efficacy based on excised specimen to anti‑PD‑1 combined with neoadjuvant chemotherapy in esophageal squamous cell carcinoma. Moreover, ICI-driven changes in the immune response are involved in peripheral blood as well as within the tumor. The most valuable markers in predicting ICI efficacy, including neutrophil-to-lymphocyte ratio (NLR) and circulating monocytes, might also implicate the peripheral blood of patients prior to and after therapy, except for immune cells within tumors [17, 18]. Additionally, several parameters in peripheral blood, such as NLR and platelet-to-lymphocyte ratio (PLR), could also be closely associated with pathological efficacy in patients with breast cancer [19] and cervical cancer [20] in the context of neoadjuvant chemotherapy. To date, in patients with ESCC who received anti-PD-1 antibody combined with neoadjuvant chemotherapy, no studies have estimated the predictive role of hematologic parameters such as NLR, PLR, lymphocyte-to-monocyte ratio (LMR), and systemic immune-inflammation index (SII) that are easier to access by physicians. Therefore, the other aim of our study was to explore whether these parameters in peripheral blood at baseline and post-treatment can predict the response to anti-PD-1 antibody combined with neoadjuvant chemotherapy in patients with ESCC.

## Patients and methods

### Patients

The data of patients with ESCC who received anti-PD-1 antibody combined with neoadjuvant chemotherapy were retrospectively collected at Sun Yat-sen University Cancer Center between June 2019 and October 2020. Patients were enrolled in our study if they met the following criteria: pathologically diagnosed ESCC, received anti-PD-1 antibody (Camrelizumab) combined with neoadjuvant chemotherapy for the first time, and were treated with Camrelizumab (200 mg) combined with taxanes including Paclitaxel or Paclitaxel for Injection (Albumin Bound), which doses are generally 135–175 mg/m^2^ and 260 mg/m^2^, respectively and platinum like Cisplatin (75 mg/m^2^) or Lobaplatin (25–30 mg/m^2^) or Fluorouracils such as Xeloda (1000 mg/m^2^, bid) and Tegafur (40 mg, bid, as less than 1.25 m^2^; 50 mg, bid, as between 1.25 and 1.5 m^2^; 60 mg, bid, as more than 1.5 m^2^) every three weeks, Three periods of treatment are generally performed before surgery, and two periods of treatment for individual patients. Exclusion criteria: 6 cases had preoperative anti-PD1 combined with neoadjuvant therapy, but the radical operation after treatment was not performed in Sun Yat-sen University Cancer Center; 1 cases had severe bone marrow toxicity after one period of treatment and were treated with granulocyte colony stimulating factor; 3 cases had preoperative anti-PD1 combined with neoadjuvant radiochemotherapy. In addition, age, sex, smoking status, stage, Body Mass Index (BMI), Eastern Cooperative Oncology Group Performance Status (ECOG-PS), tumor site, therapeutic response-based excised specimens, and blood test results at baseline and post-treatment with Camrelizumab combined with neoadjuvant chemotherapy were collected. During the treatment period, one of the 64 patients we collected had hemangioma grade I. The other 2 patients had a slight decrease in the level of blood WBC on the eighth day of the first period or platelets on the fourth day of the third period, respectively, but their treatment of three-period was performed consistently and smoothly.

### Pathological evaluation after neoadjuvant therapy

Pathological evaluation after neoadjuvant therapy was assessed using the criteria of the College of American Pathologists (CAP)/National Comprehensive Cancer Network (NCCN) [21] as follows: 0 (complete response), 1 (moderate response), 2 (mild response), and 3 (no response) under microscope by two pathologists (ZXK and LM) for all the HE slides of patients enrolled in our study.

### Parameters in peripheral blood

White blood cell count (WBC), neutrophil count (NEU), lymphocyte count (LY), monocyte count (MO) and platelet count (PLT) in peripheral blood were recorded at baseline and posttreatment. NEU divided by LY was considered as NLR, the ratio of LY to MO was considered as LMR, and PLT divided by LY was considered as PLR. Additionally, PLT multiplied by the NLR was identified as SII.

### Statistical analysis

Statistical analyses were performed using GraphPad Prism 8.0.1 and SPSS software (version 20.0; SPSS, Chicago, USA). Associations between pathological response to anti‑PD‑1 plus neoadjuvant chemotherapy and NLR, LMR, PLR, and SII at baseline and post-treatment and their cutoff values were determined by ROC curve analysis. Association between inflammatory markers and baseline characteristics was analyzed by Pearson correlation analysis. Statistical significance was defined as a two-tailed P-value <0.05.

## Results

### Clinical characteristics

The clinical characteristics of the patients are presented in Table 1. A total of 64 patients enrolled in this study consisted of 50 men and 14 women, with a median age of 62 years. There were 37 (57.8%) smokers and 27 (42.2%) non-smokers. 19 patients had clinical stage II disease and 45 patients had stage III–IV disease. All patients received the anti-PD-1 antibody plus chemotherapy, 60 of 64 patients were treated for three periods and the other 4 cases for two periods, which treatment regimens were chosen by corresponding doctors. Patients with CAP/NCCN pathological tumor regression grades 0, 1, 2, and 3 accounted for 42.2%, 21.8%, 17.2%, and 18.8% of the participants, respectively. In summary, the pathological complete response (pCR) rate was 42.2%, and the overall response rate including CAP/NCCN pathological tumor regression grades 0, 1, and 2 was 81.2%.

**Table 1:** Clinical pathological characteristics of ESCC patients receiving anti-PD1 plus chemotherapy

Characteristics (n = 64)	N (%)
Age (years)^a^	
≤ 62	34 (53.1)
> 62	30 (46.9)
ECOG-PS	
0	20 (31.3)
1	44 (68.7)
Gender	
Male	50 (78.1)
Female	14 (21.9)
Smoking	
Yes	37 (57.8)
No	27 (42.2)
T stage	
T1–T2	8 (12.5)
T3–T4	56 (87.5)
N stage	
N0	20 (31.3)
N1–N2	44 (68.7)
Clinical stage	
II	19 (29.7)
III	33 (51.6)
IV	12 (18.7)
CAP/NCCN pathological tumor regression grade	
0	27 (42.2)
1	14 (21.8)
2	11 (17.2)
3	12 (18.8)

^a^Mean age

The significant positive association was found between PLR (P = 0.012, R = 0.315) and SII (P = 0.021, R = 0.290) at baseline and ECOG-PS, and the significant negative association was showed between LMR at baseline and ECOG-PS (P = 0.010, R = − 0.320), gender (P = 0.027, R = − 0.279) and smoking status (P = 0.005, R = − 0.351). For some inflammatory makers at the post-treatment of the first period, we found that there was significant positive association between NLR and ECOG-PS (P = 0.018, R = 0.315), LMR and smoking status (P = 0.025, R = − 0.299), PLR and gender (P = 0.043, R = − 0.271), respectively. No any significant association was found between other inflammatory makers and baseline characteristics including ECOG-PS, clinical stage, age, gender and smoking status (P > 0.05, no data shown). There were no distinctly correlations were showed between clinical features (such as BMI, ECOG-PS, clinical stage, tumor site) and the status of pCR or non-pCR (P > 0.05, Table 2).

**Table 2:** The association between pCR or non-pCR status and clinical features

	pCR (n) (%)	non-pCR (n) (%)	P value
BMI			0.136
Underweight,	3 (33.3)	6 (66.7)	
Normal weight	22 (50.0)	22 (50.0)	
Overweight	2 (18.2)	9 (81.8)	
ECOG-PS			0.432
0	7 (35.0)	13 (65.0)	
1	20 (45.5)	24 (54.5)	
Clinical stage			0.742
II	9 (47.4)	10 (52.6)	
III	14 (42.4)	19 (57.6)	
IV	4 (33.3)	8 (66.7)	
Tumor site			0.945
Upper	1 (33.3)	2 (66.7)	
Middle	13 (43.3)	17 (56.7)	
Lower	13 (41.9)	18 (58.1)	

### Association between response to anti‑PD‑1 plus chemotherapy and NLR at baseline and post-treatment

When the therapeutic efficacy of patients with anti‑PD‑1 plus chemotherapy was divided into CAP/NCCN pathological tumor regression grade 0 (pCR) and grade 1, 2, 3 (non-pCR), our results seemed to show a good predictive performance for pathological tumor regression grade involving NLR at the post-treatment of the first period (AUC = 0.604, 95% CI 0.451–0.757, P = 0.183, sensitivity = 0.900, specificity = 0.385, FiguFig. 3A). NLR at baseline (AUC = 0.545, 95% CI 0.398–0.692, P = 0.548, Fig. 3B), second period (AUC = 0.518, 95% CI 0.371–0.665, P = 0.812, Fig. 3C), and third period (AUC = 0.510, 95% CI 0.355–0.665, P = 0.895, Fig. 3D) could not better predict the pathological tumor regression grade by ROC curve analysis.

**Fig. 1:**
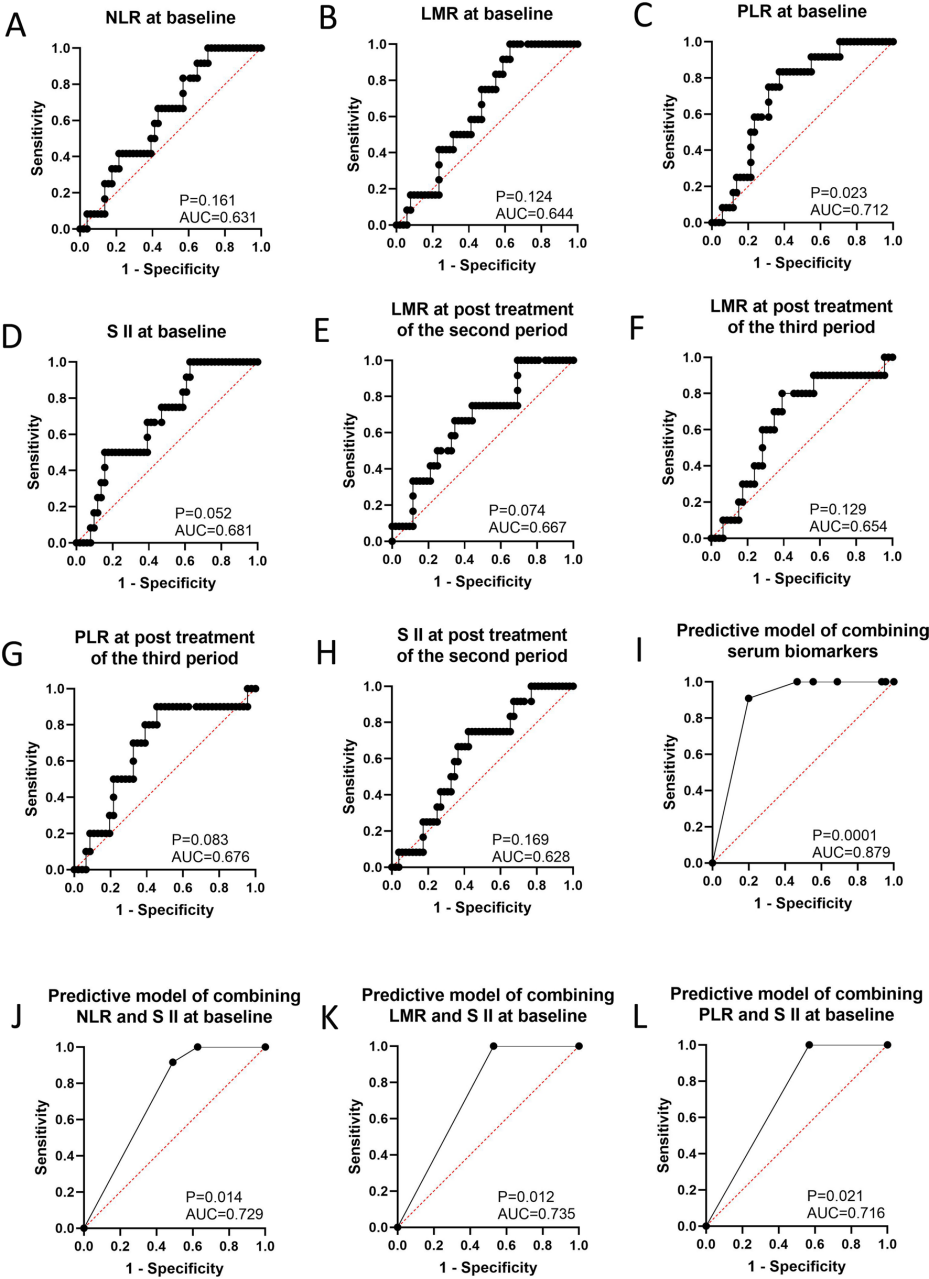
The prediction ability of serum inflammation indexes to distinguish responders and non-responders (showing the serum inflammation indexes with relatively good prediction ability). **A** NLR at baseline; **B** LMR at baseline; **C** PLR at baseline; **D** SII at baseline; **E** LMR at post treatment of second period; **F** LMR at post treatment of third period; **G** PLR at post treatment of third period; **H** SII at post treatment of second period; **I** Predictive model of combining serum biomarkers; **J** Predictive model of combining NLR and SII at baseline; **K** Predictive model of combining LMR and SII at baseline; **L** Predictive model of combining PLR and SII at baseline

**Fig. 2:**
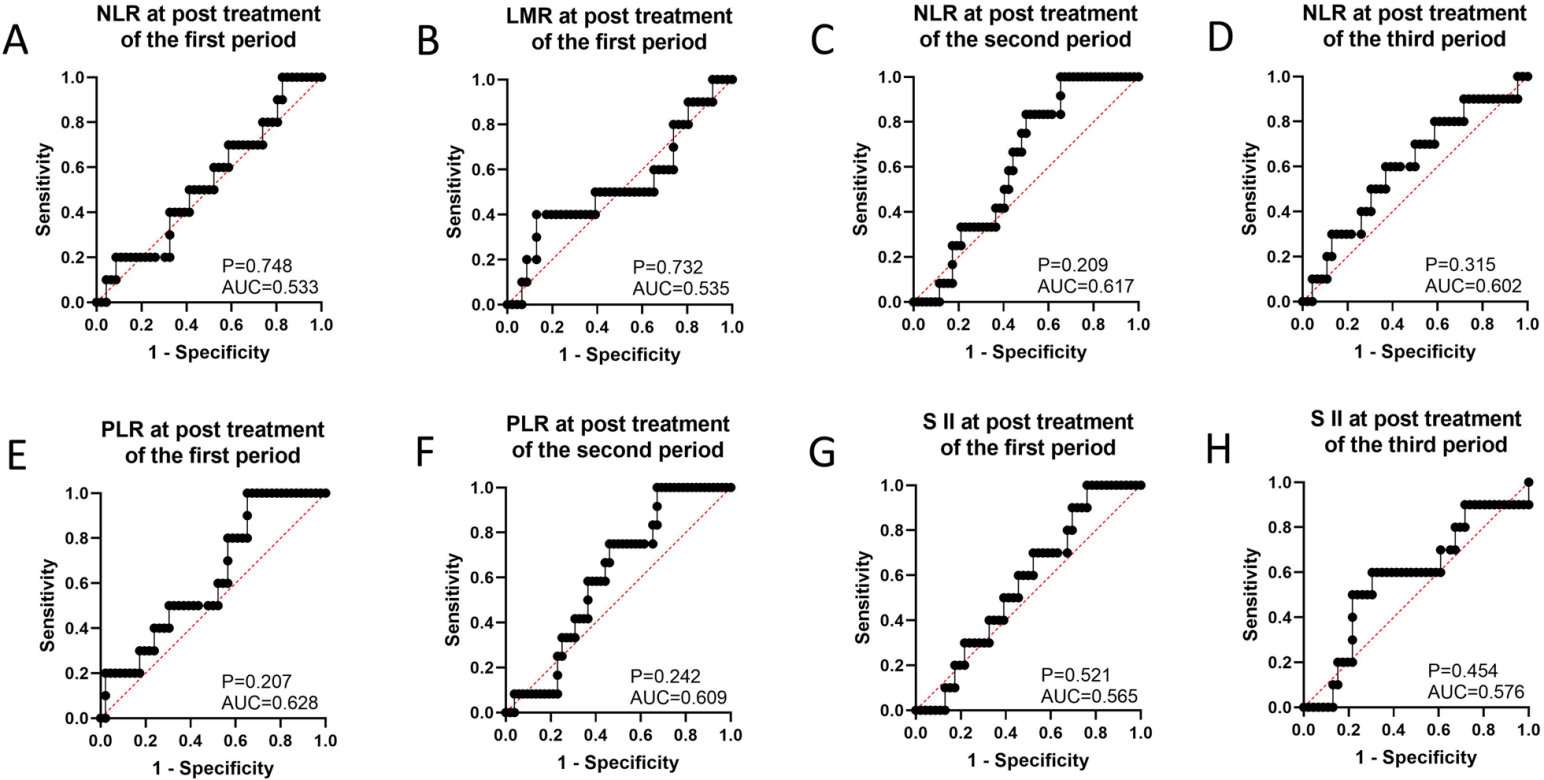
The prediction ability of serum inflammation indexes to distinguish responders and non-responders (showing the serum inflammation indexes with poor prediction ability). **A** NLR at post treatment of first period; **B** LMR at post treatment of first period; **C** NLR at post treatment of second period; **D** NLR at post treatment of third period; **E** PLR at post treatment of first period; **F** PLR at post treatment of second period; **G** SII at post treatment of first period; **H** PLR at post treatment of third period

When the therapeutic efficacy was categorized into pathological tumor regression grades 0, 1, and 2 (response) and grade 3 (no response), ROC curve analysis showed that NLR at baseline (cutoff = 1.622, AUC = 0.631, 95% CI 0.477–0.784, P = 0.161, sensitivity = 0.917, specificity = 0.137, Table 3, Fig. 1A) could be used to predict pathological tumor regression grade. However, NLR at post-treatment in the first period (AUC = 0.533, 95% CI 0.343–0.722, P = 0.748, Fig. 2A), second period (AUC = 0.617, 95% CI 0.474–0.760, P = 0.209, Fig. 2C), and third period (AUC = 0.602, 95% CI 0.409–0.795, P = 0.315, Fig. 2D) were not identified as good predictors of the pathological tumor regression grade.

**Table 3:** The number of patients with responders or non-responders corresponding to cutoff values of eight serum inflammation indexes, and clinical features

	Response (n)	No response (n)
NLR at baseline		
≤ 1.622	7	1
> 1.622	44	11
LMR at baseline		
≤ 3.173	19	0
> 3.173	32	12
PLR at baseline		
≤ 71.108	3	0
> 71.108	48	12
S II at baseline		
≤ 559.266	32	12
> 559.266	19	0
LMR at post treatment of second period		
≤ 5.987	46	11
> 5.987	6	1
LMR at post treatment of third period		
≤ 3.040	28	2
> 3.040	18	8
PLR at post treatment of third period		
≤ 151.516	22	10
> 151.516	24	1
S II at post treatment of second period		
≤ 174.574	9	1
> 174.574	43	11
BMI		
Underweight,	8	1
Normal weight	36	8
Overweight	8	3
ECOG-PS		
0	14	5
1	38	7
Clinical stage		
II	15	4
III	26	7
IV	11	1
Tumor site		
Upper	3	0
Middle	22	8
Lower	27	4

### Association between response to anti‑PD‑1 plus chemotherapy and LMR at baseline and post-treatment

**Fig. 3:**
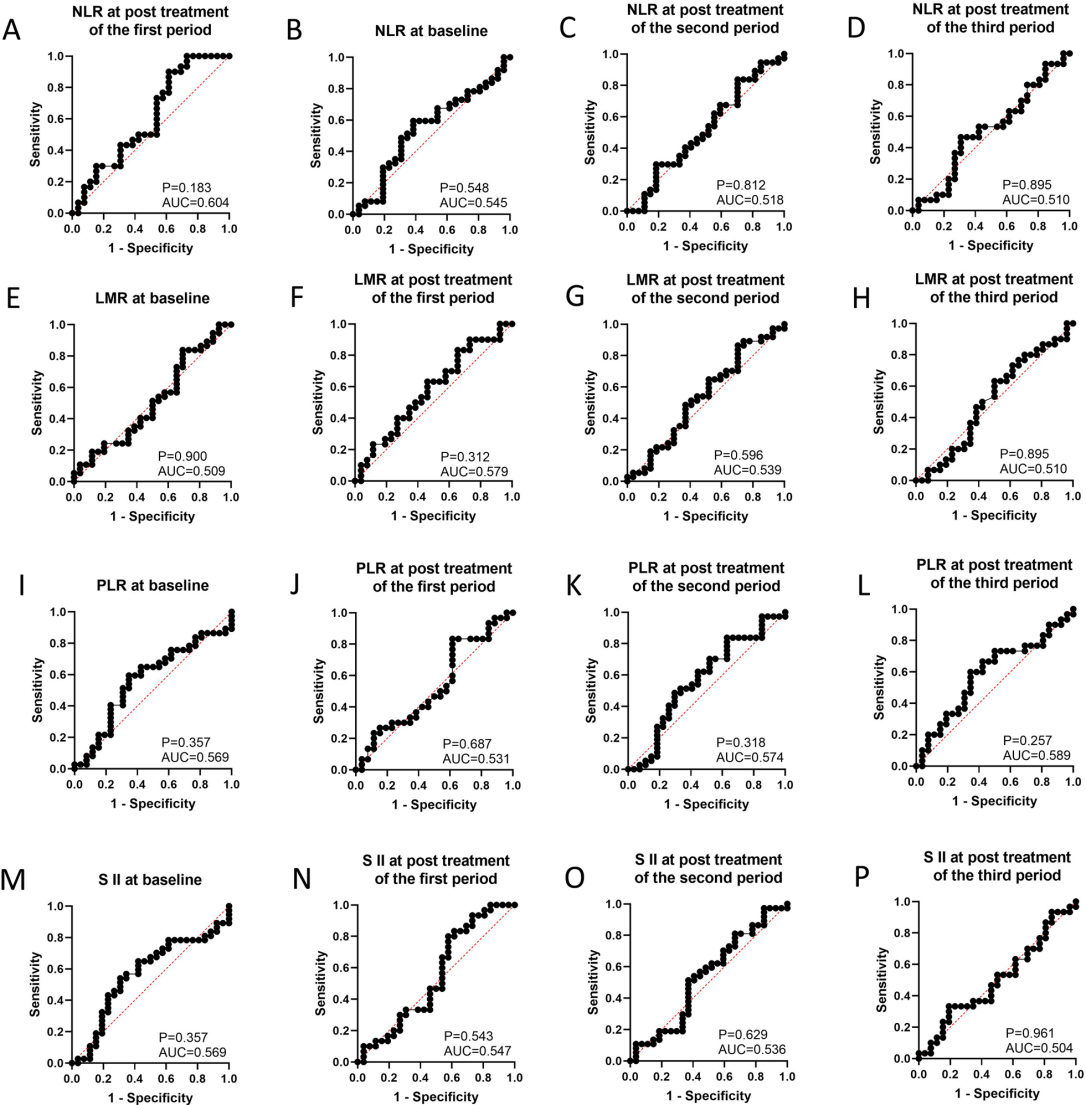
The prediction ability of serum inflammation indexes to distinguish patients with pCR and non-pCR. **A** NLR at post treatment of first period; **B** NLR at baseline; **C** NLR at post treatment of second period; **D** NLR at post treatment of third period; **E** LMR at baseline; **F** LMR at post treatment of first period; **G** LMR at post treatment of second period; **H** LMR at post treatment of third period (**I**) PLR at baseline; **J** PLR at post treatment of first period; **K** PLR at post treatment of second period; **L** PLR at post treatment of third period; **M** SII at baseline; **N** SII at post treatment of first period; **O** SII at post treatment of second period; **P** SII at post treatment of third period

ROC curve analysis showed that LMR at baseline (AUC = 0.509, 95% CI 0.361–0.658, P = 0.900, Fig. 3E) and post-treatment such as the first period (AUC = 0.579, 95% CI 0.427–0.730, P = 0.312, Fig. 3F), second period (AUC = 0.539, 95% CI 0.392–0.686, P = 0.596, Fig. 3G), and third period (AUC = 0.510, 95% CI 0.354–0.666, P = 0.895, Fig. 3H) did not significantly accurate in the prediction of the status of pCR and non-pCR.

In addition, ROC curve analysis showed that LMR at baseline (cutoff = 3.173, AUC = 0.644, 95% CI 0.500–0.788, P = 0.124, sensitivity = 1.000, specificity = 0.373, Table 3, Fig. 1B), at post-treatment of second period (cutoff = 5.987, AUC = 0.667, 95% CI 0.508–0.825, P = 0.074, sensitivity = 0.115, specificity = 0.917, Table 3, Fig. 1E) and third period (cutoff = 3.040, AUC = 0.654, 95% CI 0.476–0.833, P = 0.129, sensitivity = 0.800, specificity = 0.609, Table 3, Fig. 1F) might be useful for predicting the pathological tumor regression grade (response vs. no response). However, predictive performance of LMR at post-treatment of first period (AUC = 0.535, 95% CI 0.320–0.750, P = 0.732, Fig. 2B) was not a good predictor of the pathological tumor regression grade.

### Association between response to anti‑PD‑1 plus chemotherapy and PLR at baseline and post-treatment

The predictive performance of PLR at baseline (AUC = 0.569, 95% CI 0.422–0.715, P = 0.357, Fig. 3I) and post-treatment, such as the first period (AUC = 0.514, 95% CI 0.358–0.671, P = 0.857, Fig. 3J), second period (AUC = 0.574, 95% CI 0.426–0.721, P = 0.318, Fig. 3K), and third period (AUC = 0.589, 95% CI 0.437–0.740, P = 0.257, Fig. 3L) was shown by ROC curve analysis, and they were not good predictors for discriminating between patients with pCR or non-pCR.

Meanwhile, ROC curve analysis showed that PLR at baseline (cutoff = 71.108, AUC = 0.712, 95% CI 0.575–0.849, P = 0.023, sensitivity = 1.000, specificity = 0.059, Table 3, Fig. 1C) and post-treatment of the third period (cutoff = 151.516, AUC = 0.676, 95% CI 0.499–0.853, P = 0.083, sensitivity = 0.543, specificity = 0.900, Table 3, Fig. 1G) could effectively predict pathological tumor regression grade (response vs. no response). Moreover, the predictive performance of PLR at post-treatment in the first period (AUC = 0.628, 95% CI 0.453–0.803, P = 0.207, Fig. 2E) and second period (AUC = 0.609, 95% CI 0.461–0.757, P = 0.242, Fig. 2F) was not powerful enough to distinguish between responders and non-responders.

### Association between response to anti‑PD‑1 plus chemotherapy and SII at baseline and post-treatment

ROC curve analysis showed that the predictive performance of SII at baseline (AUC = 0.569, 95% CI 0.422–0.716, P = 0.357, Fig. 3M) and post-treatment such as the first period (AUC = 0.547, 95% CI 0.390–0.705, P = 0.543, Fig. 3N), second period (AUC = 0.536, 95% CI 0.387–0.684, P = 0.629, Fig. 3O), and third period (AUC = 0.504, 95% CI 0.350–0.657, P = 0.961, Fig. 3P) was not a good predictor of the pathological tumor regression grade (pCR vs. non-pCR).

ROC curve analysis showed that SII at baseline (cutoff = 559.266, AUC = 0.681, 95% CI 0.533–0.830, P = 0.052, sensitivity = 0.373, specificity = 1.000, Table 3, Fig. 1D) and post-treatment of the second period (cutoff = 174.574, AUC = 0.628, 95% CI 0.475–0.782, P = 0.169, sensitivity = 0.917, specificity = 0.173, Table 3, Fig. 1H) could be considered as a predictor for identifying patients with response or no response. However, SII at post-treatment in the first period (AUC = 0.565, 95% CI 0.396–0.735, P = 0.521, Fig. 2G) and third period (AUC = 0.576, 95% CI 0.377–0.775, P = 0.454, Fig. 2H) might be inappropriate to make this distinction of responders and non-responders.

Interestingly, although combining LMR with SII at post treatment of the second period could not be a good predictor for the pathological tumor regression grade (response vs. no response) (AUC = 0.567, 95% CI 0.393–0.740, P = 0.475, sensitivity = 917, specificity = 0.231), combining NLR with SII at baseline (AUC = 0.729, 95% CI 0.600–0.858, P = 0.014, sensitivity = 0.917, specificity = 0.510); LMR and SII at baseline (AUC = 0.735, 95% CI 0.609–0.861, P = 0.012, sensitivity = 1.000 specificity = 0.471); PLR and SII at baseline (AUC = 0.716, 95% CI 0.584–0.847, P = 0.021, sensitivity = 1.000 specificity = 0.431); and LMR and PLR at post treatment of third period (AUC = 0.761, 95% CI 0.605–0.917, P = 0.010, sensitivity = 0.800, specificity = 0.696) could be used to predict responders and non-responders.

### Association between response to anti‑PD‑1 plus chemotherapy and the model of combining eight serum inflammation indexes

Binary logistics analysis showed that we could combine eight serum inflammation indexes (NLR at baseline, LMR at baseline, PLR at baseline, SII at baseline, LMR at post treatment of second period and third period, PLR at post treatment of third period and SII at post treatment of second period) to construct a predictive model and screen three biomarkers including LMR at baseline, SII at post treatment of second period, and PLR at post-treatment of third period, which combination could be considered a better predictor for differentiating responders and non-responders than single or dual biomarkers (AUC = 0.879, 95% CI 0.788–0.969, P = 0.0001, sensitivity = 0.909, specificity = 0.800, Fig. 1I), and PLR at post-treatment of the third period plays an important role in the model (P = 0.030, Table 4).

**Table 4:** The logistic regression analysis of efficacy prediction for serum inflammation indexes in ESCC receiving anti-PD1 plus chemotherapy

	OR (95% CI)	P value
LMR at baseline		
≤ 3.173	1.00	
> 3.173	3.881E8 (0.000-)	0.998
S II at post treatment of second period		
≤ 174.574	1.00	
> 174.574	9.563E8 (0.000-)	0.999
PLR at post treatment of third period		
≤ 151.516	1.00	
> 151.516	0.083 (0.009–0.782)	0.030

## Discussion

Therapeutic regimens for ESCC were extremely limited until the NCT03691090 study suggested that patients could benefit from anti-PD-1 plus chemotherapy as a first-line treatment [22]. Moreover, no convenient methods have been applied to identify patients who respond to anti-PD-1 plus chemotherapy treatment. Several reports have shown that NLR, LMR, PLR, and other peripheral blood parameters correlated with the prognosis [23, 24] or radiotherapy response in ESCC [25], as well as NLR and immunotherapy response of SCLC patients [26]. The progression of gastric cancer was associated with increased interleukin-17 production by neutrophils that causes immune escape [27]. Meanwhile, circulating activated lymphocyte subsets are correlated with cancer progression [28]. These evidence indicate that peripheral blood parameters could be involved in the adaptive immune response in several cancers. Therefore, we speculated that these markers might be also identified as predictors of treatment response in patients with ESCC who received anti-PD-1 plus chemotherapy. Subsequently, our study first investigated the clinical value of NLR, LMR, PLR, and SII in predicting the response of ESCC patients to anti-PD-1 plus chemotherapy.

In this study, we found that serum inflammation indexes, such as NLR, LMR, PLR, and SII, did not accurately predict the status of pCR or non-pCR in ESCC patients. However, NLR and LMR at baseline seemed to distinguish responders from non-responders, and could accurately predict 91.7–100% of ESCC patients who had responded, but only 13.7–37.3% of patients who had no response to the agents. Meanwhile, PLR at baseline also correctly predicted 100% of responding patients, but only 5.9% of the non-responders. In contrast, SII at baseline correctly predicted 100% of non-responders and 37.3% of responders. The evidence mentioned above suggests that NLR, LMR, PLR, or SII at baseline could not be optimal biomarker for predicting responders and non-responders individually. Subsequently, we combined NLR or LMR or PLR with SII at baseline, respectively, to find that they accurately predicted 91.7–100% of ESCC patients who responded to anti-PD1 combination with chemotherapy. Furthermore, the prediction probability for patients who had no response slightly increased from 5.9–37.3% to 43.1–51.0%. To date, these inflammation indexes as prognostic biomarkers have been widely investigated in a variety of tumors, but there have been few studies on their prediction probability of agent efficacy; for example, SII at baseline could determine patients with advanced urinary tract cancer who might benefit from immunotherapy [29], and baseline NLR and LMR could predict response to first-line chemotherapy and TAS-102 + bevacizumab in advanced biliary cancer and colorectal cancer [30, 31]. In ESCC studies, meta-analysis showed that clinical indicators such as NLR, PLR, LMR, and SII had moderate predictive ability for prognosis [32], yet their prediction ability of therapeutic efficacy, especially in relation to immunotherapy, remains rarely reported.

In addition, our findings showed that LMR and SII at post-treatment in the second period could make a distinction between pathological responders and non-responders. This accurately predicted that 91.7% of ESCC patients had no response, but only 11.5–17.3% of patients responded to the agents, suggesting that combining them may result in better prediction. However, combining LMR with SII at post-treatment in the second period only increased the prediction probability to 23.1% for ESCC patients who had no response, indicating that LMR at post-treatment in the second period could not play a key role in the prediction model of LMR and SII at post-treatment in the second period. Meanwhile, LMR and PLR at post-treatment in the third period seemed to have a more balanced prediction probability for ESCC patients who had responded or no response; thus, there was a more ideal prediction probability in combining LMR with PLR at post-treatment in the third period. Prior studies reported that SII at baseline might predict pCR status for neoadjuvant chemoradiotherapy in patients with locally advanced rectal cancer [33], and non-small cell lung cancer patients with preoperative high SII levels benefit from adjuvant chemotherapy [34], which suggests a certain degree of consistency with our results. However, we attempted to combine these inflammation indices to determine whether they could predict the efficacy, instead of using a single or dual biomarkers. Finally, we aimed to construct a model consisting of eight serum biomarkers previously mentioned by binary logistics analysis and screen three of them including LMR at baseline, SII at post treatment of second period and PLR at post treatment of third period. A combination of three biomarkers could be considered a better predictor for determining responders or non-responders than any single or dual biomarkers, and maintained a good prediction probability of 90.9% for ESCC patients who responded and greatly improved the prediction value to 80% for ESCC patients who had no response. Therefore, we might use this model to screen possible responders and non-responders, which could determine follow-up treatment of these patients. We predicted that 90.9% of responding patients could continue the therapeutic regimens without surgery and 80% of ESCC patients who are non-responders truly need to undergo surgery.

Our study had some limitations. First, this was a retrospective analysis of a small sample from a single center. Second, some bias and confounding factors are inevitable, which necessitates external validation in future study due to the current rare cases. Finally, the basic biological and immune mechanisms with regard to these inflammation indexes have not been thoroughly elucidated, which somewhat explains the controversial results gotten from different studies.

In summary, the models we constructed allowed for the accurate efficacy stratification of ESCC patients receiving anti-PD-1 plus chemotherapy and is easily applicable for clinical practice at no additional cost. Future work will assess its predictive performance by external validation, and its application might help physicians predict treatment response in ESCC patients receiving anti-PD-1 plus chemotherapy in clinical practice.

## Data Availability

Derived data supporting the findings of this study are available from the corresponding author on request.
